# Diet analysis in piscivorous birds: What can the addition of molecular tools offer?

**DOI:** 10.1002/ece3.2790

**Published:** 2017-02-23

**Authors:** Johannes Oehm, Bettina Thalinger, Stephanie Eisenkölbl, Michael Traugott

**Affiliations:** ^1^ Institute of EcologyUniversity of InnsbruckInnsbruckAustria

**Keywords:** cormorant, feces, fish remains, pellet, *Phlacrocorax carbo sinensis*, prey detection, seabird

## Abstract

In trophic studies on piscivorous birds, it is vital to know which kind of dietary sample provides the information of interest and how the prey can be identified reliably and efficiently. Often, noninvasively obtained dietary samples such as regurgitated pellets, feces, and regurgitated fish samples are the preferred source of information. Fish prey has usually been identified via morphological analysis of undigested hard parts, but molecular approaches are being increasingly used for this purpose. What remains unknown, however, is which dietary sample type is best suited for molecular diet analysis and how the molecular results compare to those obtained by morphological analysis. Pellets, feces, and regurgitated fish samples of Great Cormorants (*Phalacrocorax carbo sinensis*) were examined for prey using both morphological hard part analysis and molecular prey identification. The sample types and methods were compared regarding number of species detected (overall and per sample) as well as the prey species composition and its variability among individual samples. Via molecular analysis, significantly higher numbers of prey species were detected in pellets, feces, and fish samples. Of the three sample types, pellets contained the most comprehensive trophic information and could be obtained with the lowest sampling effort. Contrastingly, dietary information obtained from feces was least informative and most variable. For all sample types, the molecular approach outperformed morphological hard part identification regarding the detectable prey spectrum and prey species composition. We recommend the use of pellets in combination with molecular prey identification to study the diet of piscivorous birds.

## 1 Introduction

Seabirds and piscivorous birds are top predators in aquatic food webs (Steinmetz, Kohler, & Soluk, 2003; Zydelis & Kontautas, 2008), and knowing their diet is important to understand their functional role in trophic networks. To determine the prey of these birds, a variety of approaches and dietary sample types can be used and examined for food remains. Videography and animal‐borne cameras are applicable to study foraging strategy and behavior of marine predators under water. However, usually only a small number of individuals can be logged or recorded, the methods are time‐consuming, prey species cannot always be identified to species level, the study period is limited to a short time span (e.g., Machovsky‐Capuska, Vaughn, Würsig, Katzir, & Raubenheimer, 2011; Machovsky‐Capuska et al., 2016; Watanabe & Takahashi, 2013; Watanuki et al., 2008), and the equipment might change the foraging performance of the observed predators (Grémillet, Enstipp, Boudiffa, & Liu, 2006). Studying fatty acid and isotope signatures of predator tissue gives a general and long‐term trophic ecological overview of the taxonomic groups or trophic levels the predators are consuming (e.g., Bearhop et al., 1999, 2006; Iverson, Field, Don Bowen, & Blanchard, 2004; Iverson, Springer, & Kitaysky, 2007; Raclot, Groscolas, & Cherel, 1998), but it does not allow for accurate prey identification on species level or the detection of short‐term changes in diet. Although taking blood, muscle, or fat tissue for these analyses is not necessarily lethal for the animals, it usually requires permission as it is stressful to them and therefore deemed invasive.

The characteristics of a wide range of dietary sample types have been evaluated and reviewed previously (Barrett et al., 2007; Carss & Group, 1997; Duffy & Jackson, 1986), including the pros and cons of both invasive and noninvasive approaches of diet analysis. Stomach content analysis is commonly used but certainly invasive as it requires shooting or catching the birds, which is not always applicable and/or possible. Therefore, sample types which can be obtained noninvasively such as pellets, feces, and dropped or freshly regurgitated fish are often preferred. The abovementioned reviews compared these sample types regarding their use for prey identification based on conventional morphological analysis of prey remains. Molecular tools applied to dietary samples have therein not been covered, although Barrett et al. (2007) recommend combining biochemical methods with conventional sampling as they can complement each other. Moreover, Carss and Group (1997) suggest that work on techniques for assessing cormorant diet should be updated as soon as new techniques such as prey‐specific molecular markers are available.

DNA‐based methods have already been successfully applied to identify prey from pellets, feces, and stomach contents of avian piscivores such as penguins (Deagle, Chiaradia, McInnes, & Jarman, 2010; Deagle et al., 2007; Jarman et al., 2013), puffins (Bowser, Diamond, & Addison, 2013), shearwaters (Alonso et al., 2014), cormorants (Oehm, Thalinger, Mayr, & Traugott, 2016; Thalinger et al., 2016), and kingfishers (Thalinger et al., 2016). Furthermore, morphological and molecular methods of prey identification have been compared in previous studies: while molecular approaches usually were found to allow detecting a wider spectrum of prey species compared to morphological analysis (Casper, Jarman, Gales, & Hindell, 2007; Deagle et al., 2010; Thalinger et al., 2016), morphological identification of prey remains provides data on prey number and size (Emmrich & Düttmann, 2011; Zijlstra & Vaneerden, 1995) which is hard to get using DNA‐based techniques. However, it remains unknown which of the noninvasively obtainable sample types—pellets, feces, and regurgitated fish—is best suited for molecular diet analysis and how the molecular results compare to those obtained by morphological analysis.

Pellets are produced regularly by seabirds and can be found at roosting and nesting sites. They contain undigested hardened remains (further on “hard parts”) of prey consumed the previous day (Zijlstra & Vaneerden, 1995; McKay, Robinson, Carss, & Parrott, 2003; but also see review in McKay, Robinson, Carss, & Parrott, 2003) and have been primarily used as a source for morphological analysis of prey hard parts. Pellets have also been found to be a viable source of prey DNA which can be identified via molecular tools (Thalinger et al., 2016).

Fecal material is often available in plenty at roosting sites of seabirds. Usually, seabirds do not produce solid scats but spout out liquid feces which can make sample collection difficult. Often it is impossible to assign a fecal sample to a single defecation event as the birds scatter their feces across the roosting site. Furthermore, as feces contain only few prey hard parts, their use in morphological diet analysis is limited (Johnson & Ross, 1996). The prey DNA, which can also be present in the fecal material, however, can make feces a valuable source for dietary information when analyzed molecularly (Deagle et al., 2007, 2010; Oehm, Juen, Nagiller, Neuhauser, & Michael, 2011).

Regurgitated fish represent the third sample type which can be obtained in a noninvasive manner as many seabirds regurgitate stomach content when disturbed or when feeding their chicks (e.g., Barrett et al., 2007; Rutschke, 1998). These prey items can be found predominately at breeding sites. Albeit relatively easy to identify morphologically, regurgitated fish might represent only part of the most recently ingested meals. In case predigested bits and pieces of fish are regurgitated, morphological identification is challenging to impossible.

In this study, pellets, feces, and regurgitated fish of seabirds were compared for the dietary information they can provide when analyzed by both morphological and molecular approaches of prey identification. We hypothesized that in all three sample types, the molecular approach will result in a larger number of prey taxa detected per sample compared to morphological identification of prey hard parts. We also predicted that a wider prey spectrum is retrievable from pellets than from feces and regurgitated fish samples. Therefore we assumed that, for a given number of dietary samples, molecularly analyzed pellets will provide the highest number of detectable prey species. Additionally, we examined the differences in prey species composition and its variability among individual dietary samples for the three different sample types.

## 2 Materials and Methods

### 2.1 Study site and collection of dietary samples

From March to August in both 2012 and 2013, representing two breeding seasons, in total, 588 regurgitated pellets, 192 feces, and 233 fish samples were collected. Collections took place every second week underneath nesting trees of Great Cormorants (*Phalacrocorax carbo sinensis*) at the shore of Chiemsee (Bavaria, Germany). The breeding colony consisted of two subcolonies (No 47.862839, E 12.503541 and No 47.859971, E 12.509115), both of them located in the estuary of the river Tiroler Achen, the main inflow of the lake. The day before sampling, horticultural cellulose fleeces were placed underneath occupied nests. The next morning, feces found on these fleeces were collected by scratching them with plastic spoons into 2‐ml reaction tubes; feces with apparently low content of white uric acid were preferably collected. Fresh pellets and fish samples found on or close to the fleeces were picked up using small plastic bags. During all collections, fresh materials (e.g., spoons and bags) were used for each sample to avoid cross‐contaminations. All samples were stored in a cooling box and transferred to the laboratory within less than 5 hr upon collection where they were kept at −32°C until further analysis.

### 2.2 Molecular analysis

For lysis, pellets and feces were defrosted and transferred into 50‐ml Greiner tubes and 2‐ml reaction tubes, respectively. Only feces collected during 2012 were subjected to further analysis. Regurgitated fish and fish parts that could not be identified morphologically using fins, coloration, and scales were thawed, and muscle tissue samples were taken using flamed forceps and scalpels for molecular identification. A buffer solution composed of TES buffer and Proteinase K (20 mg/ml) in the ratio of 190: 1 was mixed and, depending on sample size, 3–8 ml for pellets and fish tissue and 300 or 800 μl for feces were added to each sample. All samples were vortexed and incubated for at least 6 hr at 56°C. Afterward, 1.5 ml of each sample lysate was transferred into a new reaction tube which was further processed for molecular analysis. The remaining lysate was subjected to morphological analysis.

For DNA extraction, the QIAGEN BioSprint 96 instrument (QIAGEN, Hilden, Germany) in combination with the extraction protocol and reagents of the Biosprint 96 DNA blood Kit (QIAGEN) was used. Per run, 92 lysates were processed together with four extraction negative controls (TES buffer instead of lysate). Extracted DNA was eluted in 200 μl TE elution buffer and transferred into new reaction tubes which were stored at −32°C.

The molecular identification of fish based on the DNA extracts was performed by diagnostic multiplex PCR (Thalinger et al., 2016). This system covers a multitude of prey fish species occurring in the study area, that is, the home range of the cormorants inhabiting the colony at Chiemsee. It consists of six multiplex PCR assays and permits the detection of 31 fish species, six genera, two families, two orders, and two fish family clusters via the amplification of mitochondrial DNA fragments between 77 and 405 bp. In the first PCR (“FishTax”), each fish species is assigned to one of nine target groups. Four in the Alpine foreland genetically distinct species (*Acipenser ruthenus, Anguilla anguilla, Lota lota, Esox lucius*) are already identified with this assay. For the species‐rich Salmoniformes (“SalForm”), Cypriniformes (“CypForm 1‐3”), and Percomorphaceae (“PercMorph”), follow‐up PCRs enable the species‐specific identification after the taxonomically superordinate detection with the FishTax assay. The Multiplex PCR Kit (QIAGEN) was used for the 10 μl PCRs containing 1.5 μl (FishTax) or 3.2 μl (all other assays) of DNA extract. Each reaction consisted of one‐time reaction mix, 5 μg BSA, 30 mmol/L TMAC, primers in respective concentrations (Thalinger et al., 2016), and PCR‐grade water (FishTax assay only). Optimized thermocycling conditions were 15 min at 95°C, 35 cycles of 30 s at 94°C, 90 s at 64°C (FishTax, SalForm, PercMorph, CypForm 2) or 66°C (CypForm 1, CypForm 3), 1 min at 72°C, and 10 min at 72°C once (*cf*. Thalinger et al., 2016). In PCR, each 96‐well plate contained four positive controls (i.e., a DNA mix of all fish species targeted by the respective multiplex PCR assay) and two negative controls (molecular grade water instead of DNA) to check for amplification success and DNA contamination, respectively. PCR products were visualized with an automatic capillary electrophoresis system (QIAxcel; QIAGEN), the QIAxcel DNA Screening Kit (2400; QIAGEN), and its associated software (Qiaxcel Biocalculator version 3.2; QIAGEN). Samples were analyzed using the method AL320 and deemed positive, when a threshold of 0.1 relative fluorescence units was reached at the respective diagnostic amplicon sizes. All extraction negative controls were tested in PCR with the FishTax assay (Thalinger et al., 2016) and resulted negative, as well as all PCR‐negative controls.

### 2.3 Morphological analysis of prey remains

Pellets were sieved (0.5‐mm mesh size) and rinsed with water to remove soft parts and mucosa. The remaining hard parts were analyzed for otoliths, pharyngeal bones, chewing pads, and jaws used for identification. The number of fish individuals per pellet was determined by counting of otoliths and eye lenses. Fish prey remains were identified using identification keys from Härkönen (1986), Veldkamp (1995), Knollseisen (1996) as well as reference collections provided by Werner Suter (Swiss Federal Research Institute, Birmensdorf, Switzerland), Josef Trauttmansdorff (Otto König Institute, Stockerau, Austria), and the Bavarian State Collection of Zoology (Munich, Germany).

Regurgitated fish and fish parts were identified to the lowest taxonomic level possible by the diagnosis of the external morphological characteristics (Kottelat & Freyhof, 2007). If identification was not possible to species level, the fish heads, if present, were dissected and otoliths, pharyngeal bones, or chewing pads were removed for identification. If this did not permit identification, the fish sample was subjected to molecular analysis.

### 2.4 Statistical analysis

Fish prey detection rates obtained from the three sample types using molecular or morphological analysis and each fish species’ share of the total number of detected individuals resulting from morphological analysis of the pellets were calculated using MS Excel 2010 (Microsoft). Sample‐based rarefraction curves were calculated using EstimateS Version 9.1.0 (Colwell, 2013) with 500 randomized runs without replacement and extrapolation for feces and fish samples to 588 samples. Graphs were plotted using SigmaPlot 12.0 (Systat Software, Inc.) which was also used for calculations of chi‐square tests including Yates corrections testing for differences in order‐level prey detection between molecular and morphological analysis for each sample type.

As a statistical test for differences in variability/dispersion (i.e., the variation in co‐occurrence of species) between sample types, multivariate dispersion was tested using the betadisper routine in the R (R Development Core Team, 2015), package “vegan” (Oksanen et al., 2016), implementing the “permdisp2” routine (Anderson, 2006). Per sample type, the centroid of multivariate dispersion was depicted. Additionally, PERMANOVA (9,999 permutations) was used to test for significant differences in average prey species composition between the sample types.

## 3 Results

**Figure 1:**
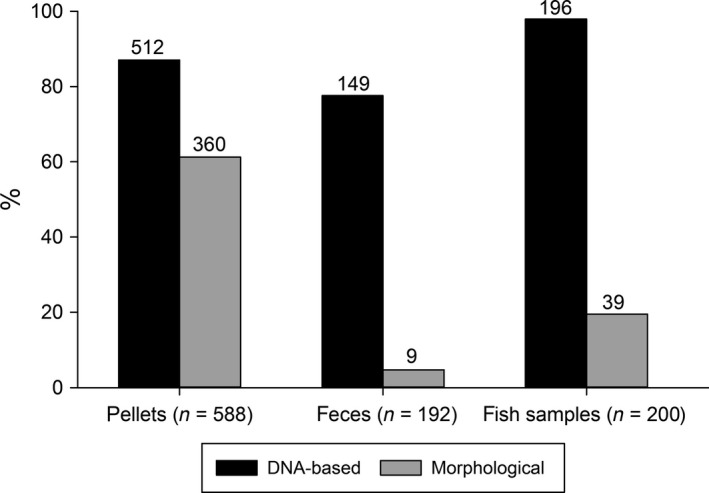
Percentage of pellets, feces, and fish samples (whole fish not included) where fish remains could be identified at least to order level using either morphological or molecular prey identification. Numbers above bars denote per sample type and method the number of positives

**Figure 2:**
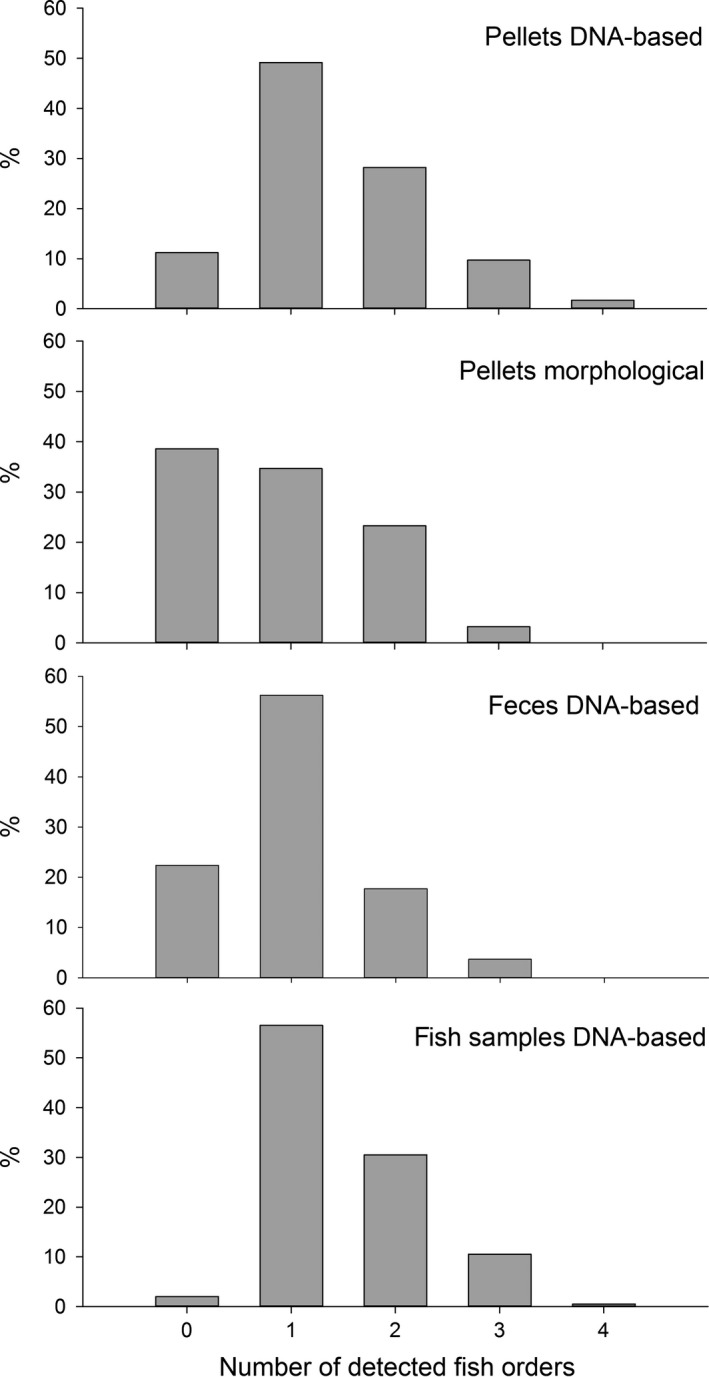
The percentage of samples containing 0–4 fish orders is graphed for each sample type (pellets, feces, fish samples) and the respective method of analysis (DNA‐based and morphological). Please note that per sample type percentages add up to 100%

**Figure 3:**
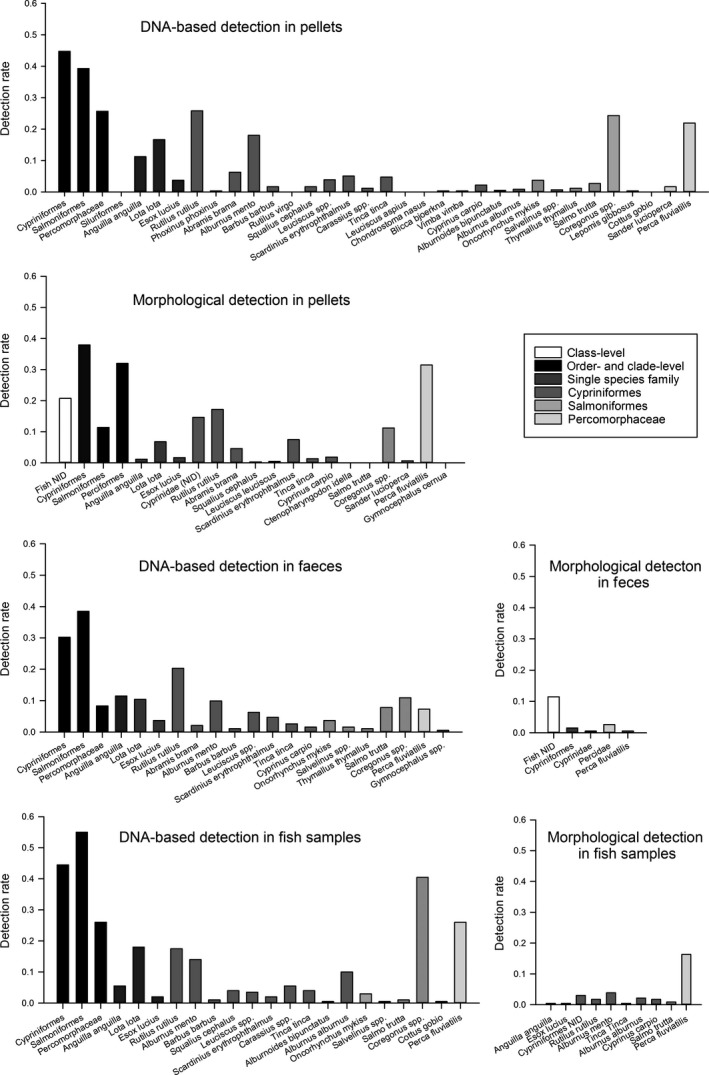
DNA‐based and morphological detection rates per prey taxon for pooled pellets, feces, and fish samples collected in a cormorant colony at Chiemsee (Bavaria, Germany) during two breeding seasons. Color codes for different taxonomic levels of identification and affiliation in Alpine foreland freshwaters; detections at the order level can result from more than one taxon per sample; thus, species and genus detection rates do not add up to order‐level detection rates

In total, 1,013 dietary samples of cormorants could be collected and analyzed during the two breeding seasons; for details on sample numbers and detections, see Table 1. Using the molecular approach, a significantly higher number of pellets tested positive for fish at order level compared to morphological prey identification (26%, χ^2^ = 101.15, *p* < .001, Figure 1), which also applied to feces (73%, χ^2^ = 207.78, *p* < .001, Figure 1), and fish samples (79%, χ^2^ = 251.05, *p* < .001, Figure 1). While the percentage of samples with fish detections (order level at least) was high for all sample types using molecular methods (pellets 87%, feces 78%, fish samples 98%), the morphological approach allowed for identifications in 61%, 5%, and 20% of pellets, feces, and fish samples, respectively. Regardless of sample type, dietary samples most frequently contained prey remains from only one fish order (pellets 49%, feces 56%, fish samples 57%); nevertheless, pellets and fish samples both contained up to four fish orders per sample (Figure 2). Within pellets, feces, and fish samples morphologically 17, 4, and 10 taxa and molecularly 31, 18, and 19 taxa, respectively, could be identified. Combining the results of DNA‐based and morphological prey detections, 33 prey fish taxa were detected in cormorant pellets compared to 18 and 21 taxa found in feces and fish samples, respectively. To avoid double‐counting, order‐ and class‐level detections of Cypriniformes, Salmoniformes, and Percomorphaceae which were genus‐ and species‐specifically identified via molecular analysis were excluded from that count. While DNA of Salmoniformes was detected most frequently in feces and fish samples, the DNA of Cypriniformes was detected most frequently in pellets. In all sample types, Cypriniformes detections consist of various cyprinid species; however, *Coregonus* spp. and *Perca fluviatilis* were by far the most often detected species of Salmoniformes and Percomorphaceae, respectively (Figure 3).

**Table 1:** For pellets, feces, and regurgitated fish, the total number of analyzed samples is displayed followed by the number of samples containing any hard parts and the fish individuals which were morphologically identifiable to order level or lower. Finally, the number of samples testing positive for fish DNA is noted per sample type. Note that for regurgitated fish samples, identifications were also made based on soft remains such as tissue

	Pellets	Feces	Fish samples
Sample number	588	192	233
Morphological			
Samples containing hard parts	455	31	38 (33 whole fish + 5 vertebrae)
Identifiable fish individuals	2,319	9	72 (33 whole fish + 39 IDs from tissue)
Molecular			
Positive for fish DNA	512	149	196 (excluding whole fish)

**Figure 4:**
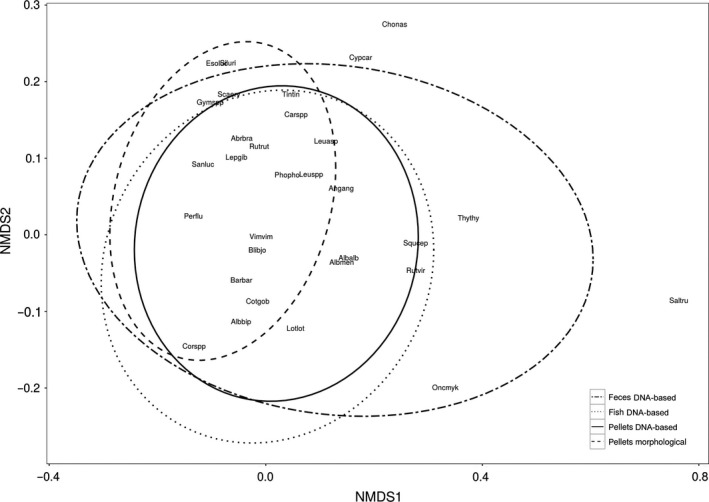
Nonmetric multidimensional scaling plot (NMDS plot, stress = 0.080) of prey species composition in pellets, feces, and fish samples of cormorants collected during breeding seasons 2012 and 2013 at Chiemsee and identified using either morphological (pellets) or molecular (all sample types) analysis. The standard ellipsoid of each sample type represents the variation (95% confidence region) in prey composition, that is, the smaller the area within the ellipsoid, the less variation in prey species co‐occurrence is found in the sample type. Fish taxa are labeled with abbreviations of their scientific name: Abrbra: *Abramis brama*, Albalb: *Alburnus alburnus*, Albmen: *Alburnus mento*, Albbip: *Alburnoides bipunctatus*, Angang: *Anguilla anguilla*, Barbar: *Barbus barbus*, Blibjo: *Blicca bjoerkna*, Carspp: *Carassius* spp., Chonas: *Chondostroma nasus*, Corspp: *Coregonus* spp., Cotgob: *Cottus gobio*, Cypcar: *Cyprinus carpio*, Esoluc: *Esox lucius*, Gymspp: *Gymnocephalus* spp., Lepgib: *Lepomis gibbosus*, Leuasp: *Leuciscus aspius*, Leuspp: *Leuciscus* spp., Lotlot: *Lota lota*, Oncmyk: *Oncorhynchus mykiss*, Perflu: *Perca fluviatilis*, Phopho: *Phoxinus phoxinus*, Rutrut: *Rutilus rutilus*, Rutvir: *Rutilus virgo*, Saltru: *Salmo trutta*, Sanluc: *Sander lucioperca*, Scaery: *Scardinius erythrophthalmus*, Siluri: Siluriformes, Squcep: *Squalius cephalus*, Thythy: *Thymallus thymallus*, Tintin: *Tinca tinca*, Vimvim: *Vimba vimba*

The variability of the prey fish community composition (i.e., the variation in co‐occurrence of species measured by dispersion) differed significantly between the sample types and the methodology used to identify the prey (*F* = 16.67; *p* < .001; Figure 4). The least variability in prey fish species co‐occurrence was found in pellets analyzed morphologically, which also had the smallest overall number of detected prey species. The prey community composition obtained by molecular analysis of pellets also showed a comparatively small variability, although the highest number of prey species was found in molecularly analyzed pellets. The highest variability in prey species co‐occurrence was found in molecularly analyzed fecal samples. Overall, the average prey species composition analyzed by PERMANOVA differed significantly between sample types (*F* = 25.81; *p* = .001; *R* ^2^ = .064).

**Figure 5:**
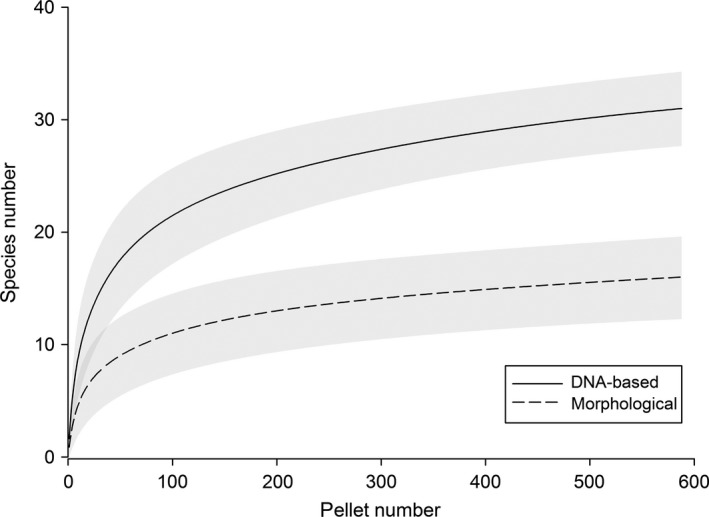
Sample‐based rarefraction curves with 95% confidence intervals comparing DNA‐based and morphological analysis of pellets with regard to species accumulation

**Figure 6:**
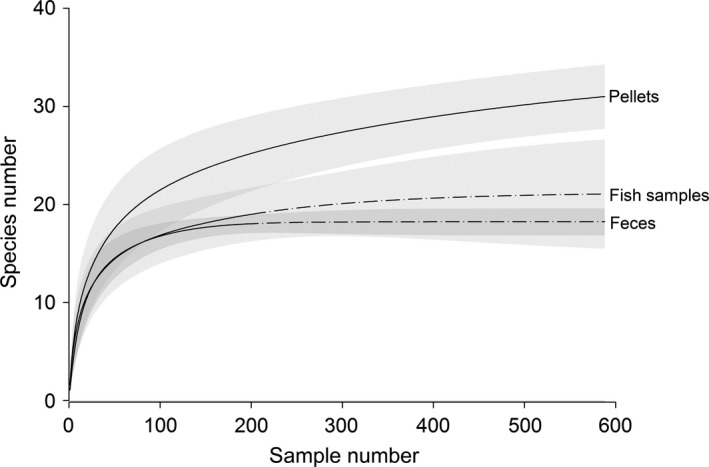
Sample‐based rarefraction curves with 95% confidence intervals comparing species accumulation between pellets, feces, and fish samples based on molecular prey detection. Note: rarefraction curves and confidence intervals were extrapolated to 588 samples for fish samples and feces

With regard to prey species accumulation, significantly more species were detected using DNA‐based pellet analysis compared to the morphological analysis from the 38th investigated pellet onwards (Figure 5). For pellets, feces, and fish samples, the comparison of species accumulation curves resulting from DNA‐based detections lead to significantly more detected species in pellets compared to feces after the 124th investigated sample. From the 238th investigated sample onwards, pellets also significantly outperformed fish samples (Figure 6).

## 4 Discussion

The present findings support our hypothesis that in all three sample types, the molecular approach results in a larger number of prey taxa detected per sample compared to morphological identification of prey hard parts. Of the three sample types investigated molecularly, 41% and 38% more fish species could be detected in pellets than in feces and regurgitated fish samples, respectively. Additionally, the rarefraction analysis of the molecularly analyzed sample types showed that at equal sample number, pellets contain the highest species number. This supports our prediction that pellets provide a wider prey spectrum than feces and regurgitated fish samples. We also found prey species composition to differ between the sample types with highest variability among individual samples in feces.

In our study, pellets proved to be the sample type that was best suited for morphological identification of fish remains (61% of pellets) as well as for the molecular detection of prey fish DNA (87% of pellets). Although a pellet should contain hard parts and DNA of all fish consumed the previous day, there were pellets which did not contain molecularly or morphologically retrievable prey remains. For prey hard parts, it is possible that they were completely digested due to an increased metabolism of stressed or juvenile cormorants (Casaux, Favero, Barrera‐Oro, & Silva, 1995; Trauttmansdorff & Wassermann, 1995; Zijlstra & Vaneerden, 1995). In 13% of the analyzed pellets also no fish DNA could be amplified. Either there was no fish uptake and therefore no fish DNA in the pellet, or the main pellet had previously been regurgitated and the regurgitation analyzed was composed of stomach mucosa only. In pellets, 15 prey species could be detected molecularly only. These species are either rare in the lake (e.g., *Leuciscus aspius*,* Rutilus virgo*,* Blicca bjoerkna*), only occur in riverine systems several kilometers away from the cormorant colony (*Chondostroma nasus*), and/or are of small size (*Phoxinus phoxinus*,* Alburnoides bipunctatus*,* Cottus gobio*). These fish are therefore rarely consumed or their prey remains strongly eroded hindering morphological identification. Interestingly, two species (*Gymnocephalus cernua* and *Ctenopharyngodon idella*) could be detected in pellets morphologically while no DNA of these species was detected in the samples. As prey hard parts are not always regurgitated in a pellet daily (McKay et al., 2003), they sometimes stay in the stomach for more than 24 hr which might leave no prey DNA to be detected on the surface of the hard parts.

Although feces have been regularly used for diet analysis of seabirds (e.g., Bowser et al., 2013; Deagle et al., 2007, 2010; Radhakrishnan, Liu, He, Murphy, & Xie, 2010), in our study, this sample type turned out to be the one where least fish taxa could be detected with both morphological and molecular analysis. Specifically for pellet‐producing seabirds, the use of feces for prey hard part analysis is limited as they contain hardly any or very small hard parts only. Molecular prey detection success strongly depends on the amount of ingested prey and on the amount of DNA surviving digestion (Deagle et al., 2007; Egeter, Bishop, & Robertson, 2015). Additionally, the content of uric acid plays an important role as this substance may inhibit DNA extraction and amplification (Deuter, Peitsch, Hertel, & Muller, 1995; Kohn & Wayne, 1997; Zarzoso‐Lacoste, Corse, & Vidal, 2013). Brownish fecal samples of cormorants were found to be preferable for dietary analysis compared to greyish feces as the latter contained high levels of uric acid and much less prey remnants (B. Thalinger, J. Oehm, M. Traugott unpublished data). Furthermore, degradation of prey DNA in feces under field conditions is an ongoing process, but the time between production and collection of the feces was kept to a minimum and the samples were not exposed to sun or rain. The average minimum temperature (roughly equates night temperatures) in July and August 2012 was 14°C only and Oehm et al. (2011) showed for feces of Carrion Crows (*Corvus corone*) that under these conditions, molecular prey detection is possible for at least 5 days. Thus DNA degradation most likely displays an insignificant role for prey DNA detection success compared to DNA content and inhibitory substances.

In 98% of the regurgitated fish samples, molecular fish detection was successful while only in 20% of the samples, hard parts revealed the identity of the fish prey. The high fish detection rate obtained via molecular analysis can be explained by the comparably fresh samples which contained less degraded DNA compared to pellets and feces. Nevertheless, in 2% of the regurgitated fish samples, no fish DNA could be amplified. This suggests that the tissue might have originated from nonfish prey as cormorants can also feed on other vertebrates such as frogs and invertebrates such as crayfish (Seefelt & Gillingham, 2006; Putys & Zarankaite, 2010; J. Oehm unpublished data). In fish samples where DNA could be amplified, up to four fish orders were detected in a single sample. This indicates that in the cormorant esophagus or stomach, the analyzed prey remain has been in contact with tissue and remnants of other prey. On the one hand, this prohibits the species‐specific identification of the regurgitated prey tissue, and on the other hand, it provides valuable information on other prey that has been consumed. This information might also be provided when hard parts of completely digested prey are regurgitated together with more recently ingested fish, but it is hardly possible to test morphologically whether the identifiable hard parts originate from the same species as the soft tissue. Hence, molecular analysis of regurgitated fish samples can provide superior information on the prey spectrum consumed together with the tissue subjected to analysis. Fish samples were, after pellets, the second best dietary sample type regarding sampling effort and prey species detected because they represent a DNA mix of several ingested prey species and undergo only little digestion.

Analyzing the pellets molecularly, predominately, Cypriniformes (*Rutilus rutilus*,* Alburnus mento*,* Abramis brama*) followed by Salmoniformes (*Coregonus* spp.) and Percomorphaceae (*P. fluviatilis*) were detected. In the same pellets analyzed morphologically, the dominant prey were Cypriniformes (mainly *R. rutilus*,* Scardinius erythrophthalmus* and *A. brama*) followed by Percomorphaceae (*P. fluviatilis*) and Salmoniformes (*Coregonus* spp.). Per sample, hard parts of fish of small to medium size were detected in high individual numbers including schooling fish such as *R. rutilus* and *P. fluviatilis*. This indicates that the investigated cormorants hunted in small flocks rather than solitary and based their diet on schooling fish of small to medium size. When a certain fish species is present in high numbers in the pellet sample, this species is more likely to be found and morphologically identified than a single individual of a potential larger fish species (e.g., *Coregonus* spp.). Furthermore, the ctenoid scales of Percomorphaceae are easier to detect and to identify (Thalinger et al., 2016) than hard parts from salmonids (McKay et al., 2003; Trauttmansdorff & Wassermann, 1995). This potential overestimation can bias the result of morphological prey detection in pellets, but can be relativized using molecular methods as they work independent of the number and condition of undigested prey hard parts.

We also examined how prey species composition varies among individual samples for pellets, feces, and regurgitated fish and found significant differences in the variability of species co‐occurrence. In pellets analyzed morphologically, the prey composition had the smallest variability of all sample types. This can be explained by the reduced number of detectable prey species (*n* = 17) which were found regularly in the pellet samples while numerous other prey species, only detected molecularly, were missed. In feces, on the contrary, there was a similar number of molecular detectable prey species (*n* = 18) but also the highest variability in the prey species composition between individual samples. This small number of species detected simultaneously per fecal sample but in manifold combinations indicates that a single fecal sample represents only a small proportion of a meal as the prey DNA from specific meals is defecated over longer time spans ranging from 2 to 48 hr in cormorants (B. Thalinger, J. Oehm, M. Traugott unpublished data) and therefore contains only a few prey species.

We found that in all three dietary sample types, molecular prey detection outperformed morphological hard part analysis regarding the detectable prey spectrum. Comparisons between molecularly analyzed samples types, however, revealed significant differences: pellets displayed molecularly the highest detectable prey spectrum with lowest sampling and analyzing effort. In feces, molecularly, the smallest number of prey species was detected and therefore a high sampling effort is needed to assess prey species richness compared to regurgitated fish samples and pellets. This should be taken into consideration when choosing the minimum sample number of feces to get a stable estimate of the trophic interaction strength between predator and prey. Furthermore, the high sensitivity of molecular tools can lead to detections of DNA from fish species which were consumed unintendedly, for example, via environmental DNA (eDNA) or secondary predation. Fish DNA of various species from the same environment can stick to ingested fish tissue or be present in the water while swallowing the fish (Rees, Maddison, Middleditch, Patmore, & Gough, 2014). This eDNA can potentially lead to false‐positive prey detections. However, DNA breakdown of this already‐fragmented eDNA is very fast and thus it is unlikely to survive digestion (Oehm et al., 2016).

The diet of cormorants breeding at Chiemsee turned out to be dominated by herbivorous and/or planktivorous fish species (*R. rutilus*,* Coregonus* spp.) or species that consume mainly invertebrates as juveniles (*P. fluviatilis*). However, piscivorous fish species such as *Anguilla anguilla*,* Esox lucius*,* P. fluviatilis*,* Sander lucioperca*,* Lota lota,* and salmonids were also frequently detected in the cormorants’ diet. Therefore, secondary predation, when a bird ingests a fish that itself recently consumed another fish, can affect the number of prey species detected. The implications of secondary predation have been discussed for molecular diet analysis (Oehm et al., 2016; Sheppard et al., 2005) as well as for morphological pellet analysis because hard parts of secondary prey may also be found in pellets (Blackwell & Sinclair, 1995). Vinson and Angradi (2011) found 25% and 40% of dissected stomachs of predatory fish to be empty, which reduces the probability of secondary predation affecting diet assessment in piscivorous birds. Moreover, Oehm et al. (2016), analyzing the stomach content of cormorants, found that in only 20% of samples where remains of predatory fish were present next to remains of potential secondarily predated prey, secondary predation could have happened based on predator–prey fish size relationships.

Generally, prey species identity and the detection frequency permit conclusions on prey–predator interactions: it shows whether and how regularly common, endangered, or invasive prey species are taken by the birds, which also displays the trophic interaction link, and indicates whether the prey was taken from the same macrohabitat (e.g., benthal or pelagial). However, as fish‐eating birds often are in conflict with fisheries, there is a high interest in quantifying mass of consumed prey species. For this purpose, morphological prey mass estimations via regression formulae based on prey hard parts are commonly used (e.g.,. Keller, 1995; Veldkamp, 1995; Gaye‐Siessegger, 2014). Unfortunately, the erosion of hard parts during digestion can bias the size estimation and therefore this method is criticized (Carss & Group, 1997). The molecular approach for prey quantification is to estimate the share of prey species in a meal via the respective DNA content. Quantitative real‐time PCR (qPCR) enables the amplification and absolute quantification of target DNA. However, the use of qPCR for targeting multiple species in one sample is technically limited to a small number and therefore this method is not appropriate for detecting and quantifying a diverse prey spectrum. Another technique is next‐generation sequencing (NGS), where a multitude of DNA fragments per dietary sample are amplified, sequenced, and assigned to the respective prey species. Trials with feces of captive marine predators showed that the amount of detected prey species DNA is not proportional to the mass of consumed prey (Deagle & Tollit, 2007; Deagle et al., 2010). Thomas, Jarman, Haman, Trites, and Deagle (2014) found the lipid content of prey to be responsible for this digestion bias and derived species‐specific correction factors to balance the mismatch. Consequently, for accurate diet proportion estimates, it would be required to determine such correction factors using control tissue of all potential prey species (Thomas, Deagle, Eveson, Harsch, & Trites, 2016), which can be very laborious and difficult to perform. In addition, digestion of captive animals might also be biased compared to digestion of wild animals so that the established correction factors do not apply to the latter. The new technique droplet digital PCR (ddPCR) allows for absolute quantification of target species DNA, but its performance for dietary investigations remains to be tested. To date, molecular tools are not ready for accurate mass quantification of consumed prey in complex ecosystems and also the number of consumed prey individuals as well as their sex and developmental stages remain furtively.

Our study compared, for the first time, the performance of molecular and morphological prey identification using three noninvasively obtained dietary sample types. It could be shown that molecular methods provide the most comprehensive dietary information, as they allow detecting more prey taxa per individual sample and a broader prey spectrum compared to morphological prey identification. This is especially true for pellets, of which least samples are required to identify the maximum detectable number of consumed prey species in comparison with feces and fish samples. As far as one of the sample types is available for a seabird species, its diet can be analyzed via molecular tools, regardless of prey species or foraging strategies. However, the molecular analysis of pellets is not inevitably the ideal approach for all types of questions regarding the feeding ecology of piscivorous birds. To enable an informed decision about the ideal sample type and best method of prey identification in future studies, we finally provide an overview on the dietary information obtainable from pellets, feces, and regurgitated fish samples using both molecular and morphological prey identification (Table 2). To investigate consumed prey species and their consumption frequency over time, molecular tools are very appropriate. For information on numbers and sizes of consumed prey individuals, morphological analysis can provide more detail; however, for comprehensive dietary studies, we recommend a combination of sample types and/or methods of analysis.

**Table 2:** Overview of samples and methods of analysis combined for noninvasively obtained dietary samples of piscivorous birds including sampling and analysis effort and information content per combination. Information is based on Duffy and Jackson (1986), Carss and Group (1997), Barrett et al. (2007), and the results obtained within the presented study

	Pellets		Feces		Fish samples	
	DNA‐based	Morphological	DNA‐based	Morphological	DNA‐based	Morphological
Sampling effort	Easily visible; collectable from soil; individual, DNA‐free sampling necessary for molecular analysis		Hardly collectable from soil; artificial surface, e.g., cellulose fleeces advisable; low urea content preferable; individual, DNA‐free sampling necessary for molecular analysis		Whole regurgitated fish easily visible and collectable; small fish parts, e.g., muscle tissue difficult to collect; individual, DNA‐free sampling necessary for molecular analysis	
Analysis effort	Lysis, DNA extraction, PCR and visualization	Washing, sieving, sorting, identification	Lysis, DNA extraction, PCR and visualization	Washing, sieving, identification	Lysis, DNA extraction, PCR and visualization	Washing, sieving, identification
Prey consumption time frame	One day; earlier ingested prey less likely detected	One day; more if hard parts are not completely regurgitated	Subsample of one meal	Subsample of one meal	One consumption plus fish DNA in the stomach at point of regurgitation	One consumption
Identification level	Species	Depending on fish family and digestive damage to hard parts; often only order level	Species	Hardly any hard parts; only distinct hard parts identifiable	Species	Species (whole fish); depending on sample: order, family, and species ID sometimes possible from fish parts
Individual count	–	Possible but not necessarily accurate	–	–	–	–
Distinct features	Best suitable for detection of complete prey spectrum.	Estimation of consumed fish individuals possible; time‐consuming	Uric acid can hamper DNA‐based analysis	Strong influence of digestion upon few detected hard parts	Species identification not always possible due to contamination in stomach	Whole fish can be further used for estimation of secondary predation

## Conflict of Interest

None declared.

## Author Contributions

BT, JO, and MT conceived and designed the study. BT, JO, and Bachelor and Master students carried out the field sampling. All molecular work was performed by BT and SE, and morphological analysis was carried out by JO and SE. BT analyzed the data and compiled tables and figures; JO wrote the manuscript which was revised and improved by BT and MT.
